# Supplementary material for: The Systems Biology Research Tool: evolvable open-source software
Source: BMC Syst Biol. 2008 Jun 29;2:55. doi: 10.1186/1752-0509-2-55 (PMC2446383; doi:10.1186/1752-0509-2-55)
Supplement: Additional file 1 — SBRT Archive. An archive of the current version of the Systems Biology Research Tool. [file 1752-0509-2-55-S1.zip › sbrt-1.4.0/doc/developers_guide/api/sbrt/kernel/utilities/proc/package-use.html]

Uses of Package sbrt.kernel.utilities.proc


|  |  |  |
| --- | --- | --- |
| **Overview** \| **Package** \| Class \| **Use** \| **Tree** \| **Deprecated** \| **Index** \| **Help** | |  |
| PREV   NEXT | **FRAMES**    **NO FRAMES**     **All Classes** |  |


---


## **Uses of Package sbrt.kernel.utilities.proc**

| Packages that use sbrt.kernel.utilities.proc | |
| --- | --- |
| **sbrt.shell.mng.utilities** | Provides classes and interfaces for creating mathematical process managers. |

Classes in sbrt.kernel.utilities.proc used by sbrt.shell.mng.utilities

****IntervalComparison****
  
          This class is used to compare sets of intervals.

****VariableParticipation****
  
          This class is used to organize a collection of mathematical expressions
according to the variables they contain.

****VectorComparison****
  
          This class is used to compare sets of intervals.

---


|  |  |  |
| --- | --- | --- |
| **Overview** \| **Package** \| Class \| **Use** \| **Tree** \| **Deprecated** \| **Index** \| **Help** | |  |
| PREV   NEXT | **FRAMES**    **NO FRAMES**     **All Classes** |  |


---
